# Periductal stromal sarcoma in a child: a case report

**DOI:** 10.1186/1752-1947-5-249

**Published:** 2011-06-29

**Authors:** Ouafae Masbah, Issam Lalya, Nawfel Mellas, Iman Bekkouch, Mohamed Allaoui, Khalid Hassouni, Tayeb Kebdani, Asmaa Regragui, Noureddine Benjaafar, Brahim Khalil Elgueddari

**Affiliations:** 1 Department of Radiotherapy, National Institute of Oncology, Allal fassi Street, Rabat 10100, Morocco; 2 Department of Medical Oncology, National Institute of Oncology, Allal fassi Street, Rabat 10100, Morocco; 3 Laboratory of Pathology, National Institute of Oncology, Allal fassi Street, Rabat 10100, Morocco; 4 Laboratory of Pathology, Agdal Oukaimden Street, Rabat 10100, Morocco

## Abstract

**Introduction:**

Periductal stromal sarcoma is an extremely rare malignant fibroepithelial tumor of the breast which is characterized by its biphasic histology with benign ductal elements and a sarcomatous stroma made of spindle cells and lacking phyllodes architecture. Its therapeutic management is based on wide surgery with free margins. Adjuvant therapies are not needed. Periductal stromal sarcoma may evolve into a phyllodes tumor with time, as well as a specific soft-tissue sarcoma. To the best of our knowledge, this tumor has never been described in a child.

**Case presentation:**

A 14-year-old Arabic boy was presented to our hospital one year ago with a nodule of the right breast that was gradually increasing in size without signs of inflammation. The histological examination after lumpectomy revealed a periductal stromal sarcoma with free surgical margins. No adjuvant treatment was given. At 50 months of close follow-up, no recurrence was observed.

**Conclusion:**

Periductal stromal sarcoma in a child is a very rare disease which has the same indolent behavior as it does in adults. Therefore, close follow-up is required.

## Introduction

Periductal stromal sarcoma (PSS) is an extremely rare neoplasm arising in the connective tissue of the breast, especially from the periductal stroma [1]. In this care report, we describe the first such case in a child reported in the literature. Diagnostic problems due to the lack of phyllodes tumors cause diagnostic problems, because PSS is a distinct, low-grade breast sarcoma with no clinical or radiological specificity. Regarding its therapeutic management, surgery with safe margins is the ideal treatment, and the efficacy of adjuvant treatment (for example, chemotherapy or radiotherapy) remains to be demonstrated [2,3].

## Case presentation

**Figure 1:**
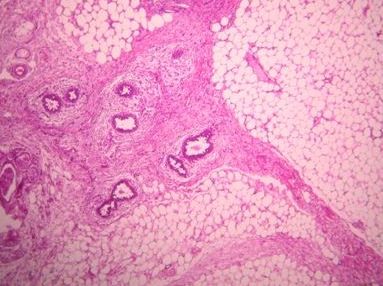
**Stromal periductal proliferation arranged within fat tissue (hematoxylin and eosin stain; original magnification, × 50)**.

**Figure 2:**
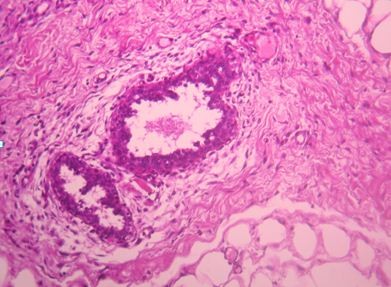
**Regular epithelial structures surrounded by a mildly cellular stroma (hematoxylin and eosin stain; original magnification, × 100)**.

**Figure 3:**
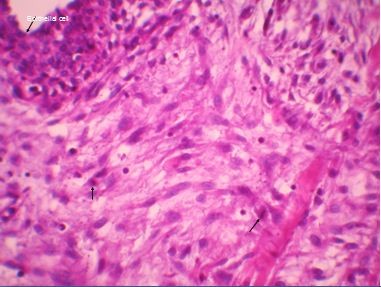
**Periductal stromal sarcoma**. Epithelial structure surrounded by a stroma with moderate atypical cells showing mitosis.

**Figure 4:**
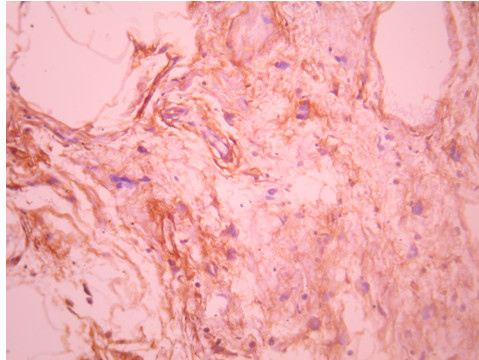
**Periductal stromal sarcoma cells are CD34-positive**.

We report the clinical case of a 14-year-old Arabic boy with no history of disease who was presented to our hospital one year ago with a nodule of the right breast that was gradually increasing in size. Upon clinical examination, we found, in the upper outer quadrant of the right breast, a small mass measuring approximately 2cm in size, round in shape, with no signs of inflammation and not associated with axillary lymph nodes. He underwent a lumpectomy. Grossly, the tumor was well circumscribed, nodular, and well delineated and measured 1.5 cm × 1 cm. Microscopic examination revealed no leafy architecture, but we found a biphasic proliferation composed of epithelial and mesenchymal components. The epithelial component corresponded to ducts with borders, sometimes double and sometimes with mild to moderate hyperplasia without atypia. These ductal structures were often dilated and surrounded by a spindle-cell mesenchymal tumor which showed moderate cellular density and mitotic activity of about two to three mitoses/10 high-power fields (Figure 1, Figure 2 and Figure 3). These mesenchymal cells showed moderately hyperchromatic nuclei of variable sizes, sometimes with distinct nucleoli. The closest distance between the tumor and resection margins ranged from 2mm to 13mm. Immunohistochemistry showed the tumor cells to be CD34-positive (Figure 4) and S-100-, ER- and PR-negative. The lesion was histologically compatible with low-grade PSS. The resection margins were negative (range, 2mm to 13mm). No adjuvant treatment was given. In a follow-up period of 50 months, the patient did not show any symptoms or signs of local or distant recurrence.

## Discussion

Previously classified with cystosarcoma with adipose metaplasia [2,4-7], PSS was recently recognized as a separate entity and was given its own place in the World Health Organization classification system at the consensus conference in Lyon in 2002 [1].

PSS occurs in pre and post menopausal women with a median of age of 55.3 years [2], and before the present case report it had never been described in a child. The symptoms most commonly found are similar to other benign and malignant breast tumors and have no radiological specificity [2].

In the presence of a breast lump in boys, clinicians must eliminate gynecomastia, which is a soft swelling or enlargement of the breast tissue under the nipple caused by the hormonal changes that occur during adolescence. Histologically, PSS is a biphasic breast tumor with benign ductal elements and a sarcomatous stroma lacking phyllodes architecture. This tumor is characterized by a hypercellular proliferation of spindle cells forming cuffs around well-preserved ductal units with infiltration of the fat and surrounding tissue. Adjacent cuffs may coalesce to form large nodules and extend into lobules surrounding open tubules and ducts. This is in contrast to mammary stromal sarcomas, which displace normal mammary tissue, entrapping ducts and lobules peripherally [3].

The histological features of PSS were defined by the Armed Forces Institute of Pathology (AFIP) [2] as follows: (1) a predominantly spindle-cell stromal proliferation of variable cellularity and atypia around open tubules and ducts devoid of a phyllodes pattern; (2) one or, more often, multiple nodules separated by adipose tissue; (3) stromal mitotic activity of ≥3/10 high-power fields; and (4) infiltration into surrounding mammary fibroadipose tissue.

Immunohistochemistry reveals the tumor cells to be positive for α smooth muscle actin CD34. They are often CD117 and do not express S-100 protein, estrogen, or progesterone receptors [2,8,9]. The histological grading depends on atypia and mitotic activity, so it ranges from being low-grade to high-grade PSS [2].

Because the number of reported cases in the literature is so small, the optimal means of managing PSS has yet to be established. Currently, resection with adequate margins is generally considered sufficient, and axillary lymphadenectomy is not needed. With regard to adjuvant therapy, the scant literature does not show any benefit of radiotherapy or chemotherapy.

PSS is a tumor of intermediate behavior; it may evolve into a phyllode tumor as well as a specific soft-tissue sarcoma. Also, PSS may occasionally exhibit intraepithelial changes ranging from ordinary hyperplasia to intraductal carcinoma [2,3]. Therefore, close follow-up is needed. Our patient is currently recurrence-free 50 months after treatment.

## Conclusion

In summary, because there is so little experience with PSS, its management remains controversial. Its histological diagnosis is based on the criteria established by the AFIP. Surgery with safe margins is the cornerstone of treatment. The prognosis of patients with PSS is unclear; thus, more cases of this unusual morphologic variant and longer follow-up of existing and future patients are needed to determine the optimal management and the clinical behavior of this neoplasm.

## Abbreviations

PSS: periductal stromal sarcoma.

## Consent

Written informed consent was obtained from the patient's next of kin for publication of this case report and any accompanying images. A copy of the written consent is available for review by the Editor-in-Chief of this journal.

## Competing interests

The authors declare that they have no competing interests.

## Authors' contributions

OM and IL contributed equally to this manuscript. OM and IL analyzed and interpreted the patient data regarding the breast disease, performed the literature research, and wrote the manuscript. NM, IB, and MA made contributions to the conception and design of the report and to the acquisition of data. KH and TK were involved in drafting the manuscript and revising it critically for important intellectual content. AR performed the histological examination of the breast and was a major contributor to the writing of the manuscript. NB and BE gave their final approval of the version to be published. All authors read and approved the final manuscript.
